# Two decades of mortality change in rural northeast South Africa

**DOI:** 10.3402/gha.v7.25596

**Published:** 2014-10-29

**Authors:** Chodziwadziwa W. Kabudula, Stephen Tollman, Paul Mee, Sizzy Ngobeni, Bernard Silaule, F. Xavier Gómez-Olivé, Mark Collinson, Kathleen Kahn, Peter Byass

**Affiliations:** 1 MRC/Wits Rural Public Health and Health Transitions Research Unit (Agincourt), School of Public Health, Faculty of Health Sciences, University of the Witwatersrand, Johannesburg, South Africa; 2 INDEPTH Network, Accra, Ghana; 3 Umeå Centre for Global Health Research, Division of Epidemiology and Global Health, Department of Public Health and Clinical Medicine, Umeå University, Umeå, Sweden

**Keywords:** Africa, Agincourt, rural, mortality, HIV/AIDS, tuberculosis, INDEPTH Network, verbal autopsy, InterVA, non-communicable diseases

## Abstract

**Background:**

The MRC/Wits University Agincourt research centre, part of the INDEPTH Network, has documented mortality in a defined population in the rural northeast of South Africa for 20 years (1992–2011) using long-term health and socio-demographic surveillance. Detail on the unfolding, at times unpredicted, mortality pattern has been published. This experience is reviewed here and updated using more recent data.

**Objective:**

To present a review and summary of mortality patterns across all age-sex groups in the Agincourt sub-district population for the period 1992–2011 as a comprehensive basis for public health action.

**Design:**

Vital events in the Agincourt population have been updated in annual surveys undertaken since 1992. All deaths have been rigorously recorded and followed by verbal autopsy interviews. Responses to questions from these interviews have been processed retrospectively using the WHO 2012 verbal autopsy standard and the InterVA-4 model for assigning causes of death in a standardised manner.

**Results:**

Between 1992 and 2011, a total of 12,209 deaths were registered over 1,436,195 person-years of follow-up, giving a crude mortality rate of 8.5 per 1,000 person-years. During the 20-year period, the population experienced a major HIV epidemic, which resulted in more than doubling of overall mortality for an extended period. Recent years show signs of declining mortality, but levels remain above the 1992 baseline recorded using the surveillance system.

**Conclusions:**

The Agincourt population has experienced a major mortality shock over the past two decades from which it will take time to recover. The basic epidemic patterns are consistent with generalised mortality patterns observed in South Africa as a whole, but the detailed individual surveillance behind these analyses allows finer-grained analyses of specific causes, age-related risks, and trends over time. These demonstrate the complex, somewhat unpredicted course of mortality transition over the years since the dawn of South Africa's democratic era in 1994.

The Agincourt Health and Socio-Demographic Surveillance System (HDSS) of the MRC/Wits Rural Public Health and Health Transitions Research Unit is an INDEPTH member site situated in the northeast of South Africa. The HDSS has been documenting mortality in a rural population since 1992. As of 2011, the population comprised some 90,000 individuals residing in 16,000 households in 27 villages (1). This paper both reviews the published outputs from Agincourt HDSS relating to mortality and presents detailed overall results based on the 20 year period 1992–2011, as contributed to the INDEPTH Network pooled cause of death analyses covering the Agincourt HDSS and 21 other INDEPTH HDSS sites (2).

Over the two decades the Agincourt site has operated, more than 40 papers have reported on particular aspects of mortality patterns and transitions, and related issues. Several papers have taken an overview of mortality at different points in time. The first signs of a reversal of mortality declines were documented at an early stage of the local HIV/AIDS epidemic (3). Subsequently, the complexities of HIV and TB co-infection were also documented (4), alongside South Africa's new struggle with increasing mortality (5). Throughout, mortality patterns have been seen to be shaped by competing forces of: 1) HIV/AIDS, 2) other communicable and nutritional diseases, 3) non-communicable diseases, and 4) violence and injuries (6, 7). Given the high proportions of HIV and TB-related mortality in this population, this has been perceived as a major issue (8–11). Maternal health and fertility, including the effects of the HIV epidemic, have also been important issues (12–14). Child mortality has been explored in relation to various risk factors (15) and adult mortality similarly (16). The relationship between cause of death and beliefs in witchcraft has also been explored (17), as well as mortality related to sleep disorders (18). Connections between mortality and migration, as geographic patterns of residence and work have gradually evolved in the post-apartheid era, have been shown to continue to be important (19–21). Finally, there has been a substantial volume of work that has capitalised on the rich detail of the Agincourt HDSS data in terms of undertaking spatio-temporal analyses of mortality patterns (22–31).

At the same time as measuring mortality patterns, the MRC/Wits Agincourt Research Unit has also served as something of a methodological incubator for mortality surveillance, starting well before the INDEPTH Network was founded in 1998. From the initial descriptions of the Agincourt HDSS and its methods (32, 33), a focus on determining cause of death became an important theme (34–37). Agincourt verbal autopsy (VA) data contributed to early work on automated cause of death assignment (38, 39). It became clear that computer-coded VA methods were indeed able to track a mortality transition as dramatic as the evolving HIV epidemic in Agincourt (40). Agincourt data again contributed substantially to the development and evaluation of the InterVA-4 VA interpretation model (41) following the release of new WHO standards for VA in 2012 (42). Pilot studies of handheld technology for undertaking VA interviews followed (43).

Against this detailed and complex background of mortality levels and trends in the Agincourt HDSS, and methodological developments in automated cause of death assignment, we present here an overview of cause-specific mortality findings over the 20-year period 1992–2011 for all age groups.

## Methods

The MRC/Wits Agincourt Research Unit has maintained individual health and socio-demographic surveillance among a rural population in the Agincourt sub-district, in northeast South Africa, starting in 1992. A detailed description of the site has been presented elsewhere (1). Annual household surveillance update rounds have identified all deaths occurring in the population, and these have been followed up with VA interviews, undertaken by specifically trained interviewers 1–11 months after death. Local physicians have assigned causes of death to the VA material, which has also more recently been processed retrospectively using the InterVA-4 probabilistic model (version 4.02) (41). As far as is possible, the VA instrument and InterVA-4 model aim to arrive at underlying causes of death, but this is not always possible with certainty using VA methods. Nevertheless, it has been established that causes of death based on results generated by the InterVA-4 model do not differ substantially from physician findings (40). In addition, the InterVA-4 model assigns causes of death in a standardised automated manner that is much quicker and more consistent than physician coding. Rates of cause-specific HIV associated mortality using the InterVA-4 model have been explored in a multisite study (44).

The outputs from the InterVA-4 model, up to three causes for each case plus a possible indeterminate residual fraction, for Agincourt contained in the INDEPTH Network Cause-Specific Mortality multisite dataset (45) have been analysed with the same age-sex-time standardisation used for the INDEPTH cross-site comparisons (46). The standardisation was necessary because the age-sex population structure in the Agincourt site changed appreciably over the 20-year period (1). Consequently, in considering changing patterns of causes of death, it is important to standardise the age-sex structure to ensure that changes observed in particular causes are not due to age-sex changes in the population over time. Because the site covers an entire defined population, it is not meaningful to ascribe confidence intervals, although individual-level uncertainty in cause of death is captured through the indeterminate component of the InterVA-4 output.

The Agincourt HDSS was reviewed and approved by the Committee for Research on Human Subjects (Medical) of the University of the Witwatersrand (protocol M960720 and M081145). Community consent from civic and traditional leadership was secured at the start of surveillance and is reaffirmed from time to time, and informed verbal consent is obtained at individual and household level at each annual follow-up visit. A record is kept of the household respondent who consented to be interviewed as well as the responsible fieldworker.

## Results

Over the period 1992–2011 a total of 12,209 deaths were registered over 1,436,195 person-years of follow-up, corresponding to a crude mortality rate of 8.5 per 1,000 person-years. After standardisation, these amounted to 13,153 deaths, of which 12,010 (91.3%) had VA interviews successfully completed. For 607 (5.1%) of these, the InterVA-4 model was unable to reach any conclusive cause of death.

**Fig. 1:**
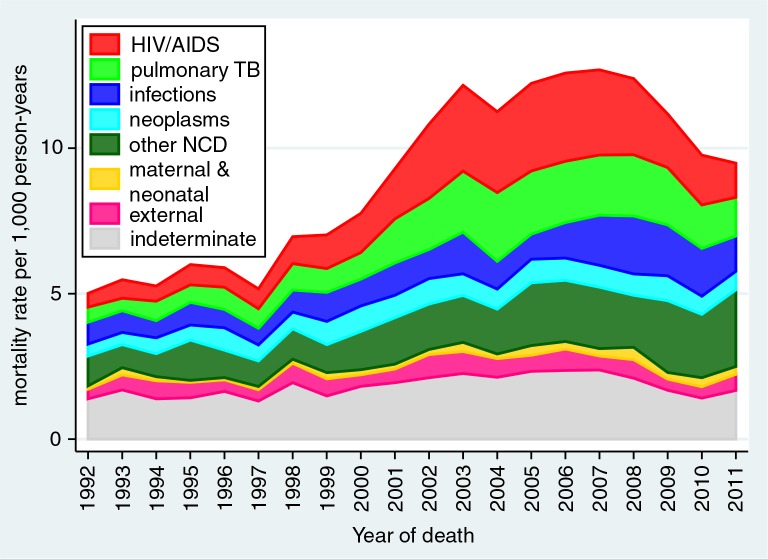
Age-sex-time standardised mortality rates by broad cause categories, Agincourt HDSS.

**Fig. 2:**
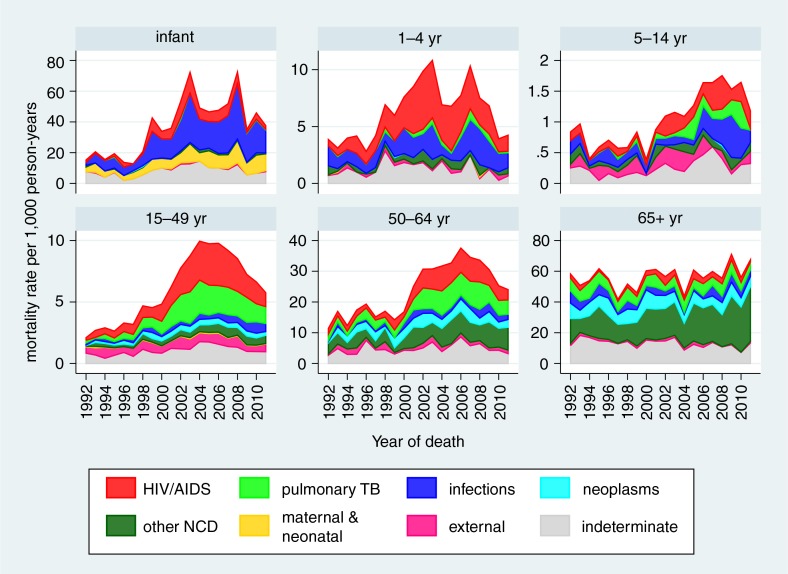
Age-sex-time standardised mortality rates by broad cause categories and age group, Agincourt HDSS.

**Fig. 3:**
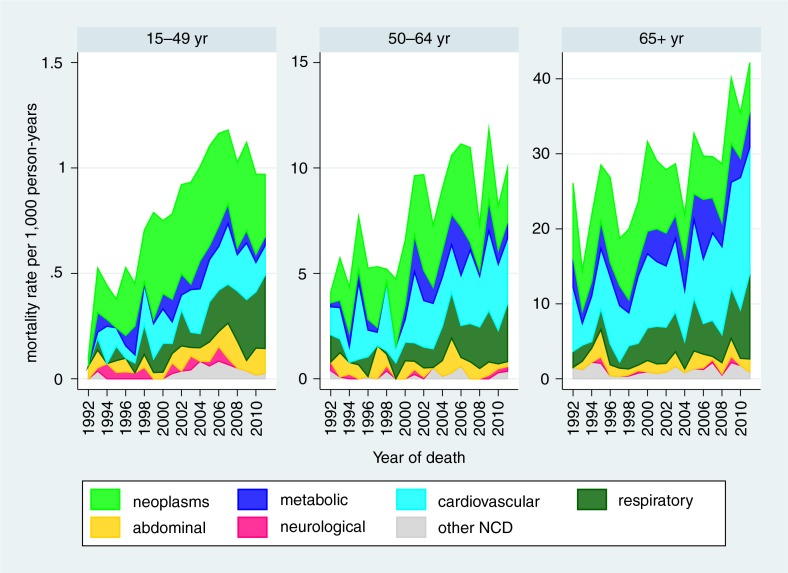
Age-sex-time standardised non-communicable disease (NCD) mortality in adult age groups, Agincourt HDSS.

Table 1 presents detailed standardised mortality rates by age group and causes of death according to the WHO 2012 VA standard categories (47). Figure 1 shows the development of standardised mortality rates by cause categories over time, clearly showing the overall mortality epidemic peak associated with HIV in this population around 2007 and reduction in the most recent time period. Figure 2 shows the same data for the separate WHO 2012 age groups (with neonatal and 1–11 month age groups combined into a single infancy category). In this figure, the vertical scales for mortality rates are appreciably different between the different age groups, with highest rates for infants and over-65 year olds, and lowest rates for the 5–14 year age group. All age groups except the over-65 year olds experienced reduction in mortality rate from around 2007. Mortality increased steadily in the over-65 year olds over the 20-year period. Figure 3 shows trends in non-communicable disease (NCD) mortality in adult age groups. NCD mortality steadily increased in all adult age groups from the late 1990s and declined in the most recent time period only in the 15–49 year age group. The increase in NCD mortality in the 50–64 and 65+ year age groups are largely driven by increases in cardiovascular conditions (including stroke and acute cardiac disease) followed by respiratory conditions (including chronic obstructive pulmonary disease). In the 15–49 year age group, trends in NCD mortality mirror trends in HIV/AIDS mortality.

**Table 1:** Age-sex-time standardised cause-specific mortality rates per 1,000 person-years by WHO 2012 causes and age group, for a total of 12,209 deaths registered over 1,436,195 person-years at the Agincourt HDSS, 1992–2011

Cause of death	Neonate	1–11 mo	1–4 yr	5–14 yr	15–49 yr M	15–49 yr F	50–64 yr	65+ yr
01.01 Sepsis (non-obstetric)		0.601	0.006	0.001	0.004	0.003	0.028	0.109
01.02 Acute resp infect, incl pneumonia		10.563	0.788	0.124	0.268	0.275	1.102	3.177
01.03 HIV/AIDS related death		5.172	2.361	0.193	1.130	2.474	4.393	2.883
01.04 Diarrhoeal diseases		4.087	0.500	0.012	0.035	0.035	0.158	0.548
01.05 Malaria		0.461	0.233	0.052	0.057	0.078	0.094	0.109
01.06 Measles		0.196	0.015	0.003				
01.07 Meningitis and encephalitis	1.849	0.196	0.068	0.009	0.020	0.023	0.024	0.011
01.09 Pulmonary tuberculosis		0.187	0.237	0.103	1.656	1.279	4.796	6.977
01.10 Pertussis		0.653	0.033					
01.11 Haemorrhagic fever				0.006	0.005			
01.99 Other and unspecified infect dis		0.121		0.018	0.021	0.013	0.097	0.212
02.01 Oral neoplasms					0.013	0.010		0.072
02.02 Digestive neoplasms					0.133	0.107	1.357	2.704
02.03 Respiratory neoplasms					0.096	0.084	0.879	2.384
02.04 Breast neoplasms						0.066	0.019	0.124
02.05, 02.06 Reproductive neoplasms M,F					0.014	0.084	0.133	0.730
02.99 Other and unspecified neoplasms		0.057	0.005	0.002	0.075	0.019	0.370	1.703
03.01 Severe anaemia							0.021	0.011
03.02 Severe malnutrition		0.321	0.214	0.005	0.005	0.002	0.037	0.361
03.03 Diabetes mellitus		0.030	0.013	0.008	0.065	0.050	0.757	3.249
04.01 Acute cardiac disease					0.023	0.006	0.204	0.355
04.02 Stroke				0.003	0.072	0.079	1.063	4.966
04.99 Other and unspecified cardiac dis			0.008	0.002	0.056	0.074	1.071	4.688
05.01 Chronic obstructive pulmonary dis					0.076	0.051	0.645	3.162
05.02 Asthma			0.090	0.034	0.058	0.110	0.626	1.444
06.01 Acute abdomen		0.375	0.083	0.010	0.045	0.054	0.357	0.913
06.02 Liver cirrhosis			0.043	0.009	0.019	0.016	0.138	0.327
07.01 Renal failure		0.016	0.006	0.004	0.013	0.003	0.070	0.208
08.01 Epilepsy			0.009	0.022	0.025	0.019	0.087	0.162
98 Other and unspecified NCD					0.036	0.026	0.169	1.204
10.06 Congenital malformation	0.753	0.294	0.015					
10.01 Prematurity	12.115							
10.02 Birth asphyxia	21.428							
10.03 Neonatal pneumonia	42.198							
10.04 Neonatal sepsis	5.590							
10.99 Other and unspecified neonatal CoD	11.196							
12.01 Road traffic accident		0.128	0.116	0.073	0.385	0.094	0.321	0.312
12.04 Accid drowning and submersion	0.384		0.015	0.036	0.011		0.015	
12.05 Accid expos to smoke, fire & flame		0.073	0.028	0.015	0.013	0.014	0.08	0.175
12.07 Accid poisoning and noxious subs			0.023		0.009	0.001	0.003	0.016
12.08 Intentional self-harm				0.014	0.103	0.122	0.174	0.115
12.09 Assault		0.077	0.008	0.010	0.479	0.119	0.533	0.345
12.99 Other and unspecified external CoD			0.019	0.013	0.005		0.047	0.138
09.01 Ectopic pregnancy						0.002		
09.02 Abortion-related death						0.001		
09.03 Pregnancy-induced hypertension						0.015		
09.04 Obstetric haemorrhage						0.053		
09.05 Obstructed labour						0.004		
09.06 Pregnancy-related sepsis						0.013		
09.07 Anaemia of pregnancy						0.013		
09.99 Other and unspecified maternal CoD						0.012		
99 Indeterminate	30.952	6.298	1.241	0.252	1.244	0.919	4.855	12.914
All causes	126.465	29.906	6.177	1.033	6.269	6.422	24.723	56.808

## Discussion

Mortality findings at the Agincourt HDSS for the overall period 1992–2011 reflect, as expected, many facets from interim publications. This paper provides a 20-year retrospective picture of a community in which mortality patterns changed drastically over time as a consequence of the HIV epidemic, as is clearly visible in Fig. 1. Few epidemics in human history have caused a sudden doubling in all-cause mortality in a particular population, sustained over a decade. There is still some way to go until mortality rates return to near pre-epidemic levels, despite encouraging recent decreases.

Attributing individual deaths unambiguously to HIV, in the absence of biomedical information, is not simple. Another study showed that the InterVA-4 model achieved a high level of specificity for HIV/AIDS related mortality in relation to sero-status (44). However, it was also clear that, given higher cause-specific mortality rates for other causes among HIV positives, substantial proportions of HIV-related mortality are likely not to be attributed as such. This is seen in the Agincourt population, with substantially higher rates of TB, other infections, and NCDs during the peak incidence of HIV/AIDS deaths. There may also be some concomitant increases in NCD mortality rates due to other risk factors such as smoking and obesity, but the INDEPTH multisite analysis of adult NCD mortality showed significant correlations between HIV and NCD mortality, over a wide range of rates (48).

Figure 2 details the effects of the HIV epidemic on each age group. Although in infancy HIV/AIDS as a specific cause was not the major cause of death, it is clear that other infections were mainly responsible for the increase in infant deaths due to the peak of the overall epidemic. It must be presumed therefore that these additional infant deaths due to other infections were associated with HIV. In the 1–4 year age group, the major increases appear to be more clearly associated with clinically typical HIV/AIDS, and hence a large part of the additional mortality was assigned as such. The mortality peaks both for the infant and 1–4 year age groups appear to slightly precede the peaks in older age groups. On the assumption that most of the childhood HIV/AIDS deaths would follow perinatally acquired infections, this may reflect young children developing disease fairly rapidly and dying from HIV-related causes before their mothers also went on to die, particularly at the stage before prevention of mother to child transmission (PMTCT) and adult anti-retroviral treatment (ART) programmes were well established in South Africa. It is not clear what would have caused the two peaks in the infant and child mortality patterns, but because they occurred during the time of peak HIV/AIDS mortality, they could have resulted from inconsistencies in the provision of PMTCT. For the 5–14 year age group, the peak of HIV-related mortality was later than in other age groups. HIV still accounted for a substantial increase in the relatively low overall mortality rates in this age group, presumably largely reflecting perinatal infections some years earlier in a sub-group of children who survived for several years. A study of mortality in young people found that HIV and TB combined was the third ranking cause of death globally in the 10–14 age group (49), and HIV/AIDS was the leading cause of adolescent hospitalisation in Zimbabwe (50).

Among adults, the 15–49 year age group had the largest proportion of HIV/AIDS deaths, though the cause-specific rate was lower than in the 50–64 year age group. In both of those age groups, rates of TB and NCDs increased substantially with the increase in HIV/AIDS, but there was no sign of NCDs decreasing in later years as HIV/AIDS and TB decreased. The 65+ year age group was the least affected by HIV/AIDS, as might be expected, although the relatively small proportion of HIV/AIDS deaths in this age group even during the epidemic peak amounted to an appreciable mortality rate, given the high overall mortality in this age group. As would be expected, NCDs accounted for the single largest category in this age group, and also increased over time.

Despite improvement since 2007, and contrary to global trends, infant and under-5 mortality rates (Fig. 2) remained substantially higher in 2011 than they were 20 years earlier. There is still some way to go to achieving reductions in these key parameters, not only in relation to HIV/AIDS, but also in terms of further improvements in the provision of maternal and perinatal health care. Inevitably the major political changes in South Africa over the same period are likely to have also affected mortality rates. However, from an analytical perspective, given the overwhelming impact of the HIV epidemic, it is hard to distinguish the effects of socio-political developments on mortality.

From a methodological perspective, despite some shortcomings associated with the use of proxy respondents (common to all VA methods), and that vital events are updated annually in Agincourt resulting in longer recall periods and potentially underestimating perinatal and infant mortality (1), the evidence is very clear that the surveillance operations mounted in the Agincourt HDSS have succeeded to a large extent in identifying and registering deaths, and subsequently determining cause of death. The use of the WHO 2012 VA standard and the InterVA-4 model, even as applied here retrospectively to VA material that was not originally envisaged to be computer coded, has resulted in a plausible picture of a major epidemic. This was possible even though the methods had no means of ‘knowing’ about the development of the epidemic. This clearly demonstrates that it is feasible to use a general model for cause of death assignment, which does not need to be fed with local knowledge of mortality patterns and changes.

The analyses in this paper were restricted by the available data, and conformed to WHO-defined age groups and cause categories. We anticipate forthcoming papers utilizing advanced statistical analysis techniques and an extended time period to capture more fully the effects of the public sector rollout of ART and also assess the impacts of the seismic political and social changes following the abolition of racial segregation (apartheid), including the changing exposures associated with evolving patterns of migration and livelihoods, and the first universal elections in South Africa in 1994.

## Conclusions

The Agincourt population has experienced a major mortality shock over the past two decades, from which it will take time to recover. The basic mortality pattern is consistent with the generalised pattern of mortality from an unfinished burden of communicable and nutritional diseases, HIV/AIDS, NCD, and violence and injuries observed elsewhere in South Africa (51–54), but the detailed individual surveillance behind these analyses allows finer-grained analyses of specific causes, age-related risks, and trends over time. The mortality trends presented here contribute to the understanding of South Africa's progress towards the achievement of some of the health-related millennium development goals. Most important, the trends highlight the importance of monitoring the evolution of NCD conditions and their risk factors and the need for South Africa to focus on their prevention, control, and treatment while continuing to strengthen HIV/AIDS prevention and treatment programmes in order to achieve further reductions in mortality rates.
